# Melatonin Modulates Neuronal Cell Death Induced by Endoplasmic Reticulum Stress under Insulin Resistance Condition

**DOI:** 10.3390/nu9060593

**Published:** 2017-06-10

**Authors:** Juhyun Song, Oh Yoen Kim

**Affiliations:** 1 Department of Biomedical Sciences, Center for Creative Biomedical Scientists at Chonnam National University, Gwangju 61469, Korea; alj1008@nate.com; 2 Department of Food Sciences and Nutrition, Dong-A University, 37 550beon-gil Nakdongdaero, Saha-gu, Busan 49315, Korea

**Keywords:** melatonin, neuroblastoma cells, insulin resistance, endoplasmic reticulum stress, cell death

## Abstract

Insulin resistance (IR) is an important stress factor in the central nervous system, thereby aggravating neuropathogenesis and triggering cognitive decline. Melatonin, which is an antioxidant phytochemical and synthesized by the pineal gland, has multiple functions in cellular responses such as apoptosis and survival against stress. This study investigated whether melatonin modulates the signaling of neuronal cell death induced by endoplasmic reticulum (ER) stress under IR condition using SH-SY5Y neuroblastoma cells. Apoptosis cell death signaling markers (cleaved Poly [ADP-ribose] polymerase 1 (PARP), p53, and Bax) and ER stress markers (phosphorylated eIF2α (p-eIF2α), ATF4, CHOP, *p-IRE1*, and spliced XBP1 (sXBP1)) were measured using reverse transcription-PCR, quantitative PCR, and western blottings. Immunofluorescence staining was also performed for *p-ASK1* and *p-IRE1*. The mRNA or protein expressions of cell death signaling markers and ER stress markers were increased under IR condition, but significantly attenuated by melatonin treatment. Insulin-induced activation of *ASK1* (*p-ASK1*) was also dose dependently attenuated by melatonin treatment. The regulatory effect of melatonin on neuronal cells under IR condition was associated with *ASK1* signaling. In conclusion, the result suggested that melatonin may alleviate ER stress under IR condition, thereby regulating neuronal cell death signaling.

## 1. Introduction

Insulin resistance (IR) is a pathological condition observed in people with type 2 diabetes mellitus (T2DM) [1], obesity, or older age [2]. Recent epidemiological and human studies have reported that IR leads to cognitive decline and dementia in the central nervous system [3,4,5,6]. Animal studies have also demonstrated that IR impaired behavioral function in high fat-fed mice [7,8]. IR is directly involved in the dysregulation of cellular homeostasis [9], and endoplasmic reticulum (ER) stress, which triggers cell death signaling [10,11]. Under stressful conditions, three ER stress receptors on the ER membrane are activated sequentially, with pancreatic ER kinase (PKR)-like ER kinase (PERK) being the first, followed by the activation of transcription factor 6 (ATF6), and with IRE1 being the last [12]. Activated PERK blocks protein synthesis by phosphorylating eIF2α [13], and the phosphorylation makes ATF4, a transcription factor, to translocate into the nucleus, and induce the transcription of genes needed to restore ER homeostasis (i.e., amino acid synthesis and transport, stress response, redox reaction, and CHOP, etc.) [14]. CHOP is a protein regulating ER stress mediated apoptosis through the modulation of pro-apoptotic and anti-apoptotic proteins [14,15], and also a cardinal mediator in IR [16], mitochondrial apoptosis [17], and inflammation [18]. Activated ATF6, a transcription factor, also regulates the expressions of ER chaperones, CHOP, and XBP1 [19]. Spliced XBP1 (sXBP1), which is an active form of XBP1 spliced by IRE1, translocates into the nucleus and controls the transcription of chaperones, P58^IPK^, and genes for protein degradation [20,21]. In fact, increased sXBP1 levels were observed in patients with metabolic diseases [22,23]. Additionally, phosphorylated IRE1 (*p-IRE1*) was reported to recruit the adaptor molecule tumor necrosis factor-receptor-associated factor 2 (TRAF2) [24]. The IRE1–TRAF2 complex formed under the ER stress can recruit an apoptosis signal-regulating kinase 1 (*ASK1*), a member of a large MAPK kinase family known to aggravate cell death [24,25,26,27,28]. That is, under persistent stress, IRE1 triggers apoptosis by recruiting ASK1. These above factors may strongly suggest that ER stress is an important therapeutic target for IR and T2DM [29,30]. Collectively, ER stress should be highlighted for the investigation on the mechanisms of neuropathogenesis caused by IR. 

Recently, melatonin (5-methoxy-*N*-acetyltryptamine) was reported to alleviate ER stress by stimulating cell survival signaling such as the Akt pathway in oxidative stress [31]. Melatonin is a pleiotropic hormone synthesized by the pineal gland, secreted into the blood stream, and involved in the entrainment of the circadian rhythm such as sleep and wake timing, blood pressure controlling, energy balance favoring and inhibiting brown adipose tissue formation, and seasonal reproduction, etc. [32,33]. It is also a phytochemical compound present in various foods from fungi to animals and plants [34]. Melatonin (and its metabolites) also plays multiple roles as a potent antioxidant and a free radical scavenger in cellular homeostasis (i.e., cell survival, apoptosis, thermogenesis, inflammation, etc.) by binding specific melatonin receptors (i.e., MT1, MT2) [35,36,37,38,39,40,41,42,43,44], and also improves the nuclear or mitochondria dysfunction in diabetic and obese animal model through its antioxidant capacity [45,46,47,48]. However, few studies have reported the regulatory effect of melatonin on ER stress-induced neuronal cell death signaling under IR condition. Therefore, this present study investigated whether melatonin modulates the ER stress under IR condition, thereby regulating neuronal cell death signaling. The results would provide a specific mechanism for how melatonin affects cell survival against IR-induced stress.

## 2. Methods and Materials

### 2.1. Cell Culture

SH-SY5Y neuroblastoma cells (ATCC, Manassas, VA, USA) are capable of differentiating into neuron-like cells in the presence of retinoic acid (RA). Undifferentiated SH-SY5Y cells were cultured at 37 °C with 5% CO_2_ in Dulbecco’s modified Eagle’s medium (DMEM) supplemented with 10% fetal bovine serum (FBS; Gibco, Grand Island, NY, USA) and 100 μg/mL of penicillin-streptomycin (Gibco). SH-SY5Y cells were passaged at least twice, and differentiated with a replacement of fresh media supplemented with 1% FBS and 5 μM of RA, and cultured in DMEM media with 100 nM of insulin (Wako Chemicals, Richmond, VA, USA) for 3 days, with a replacement of fresh medium every 24 h [17]. Afterwards, medium was replaced with serum-free DMEM media. After 30 min, cells were exposed to 1 μM of insulin for 15 min [18,19]. NQDI-1, an apoptosis signal-regulating kinase 1 (ASK1) inhibitor, was purchased from Tocris Bioscience (Bristol, UK). Cells were pretreated with *ASK1* inhibitor (600 nM) for 2 h to inhibit *ASK1* activation before IR stress.

### 2.2. Cell Viability Assay

SH-SY5Y cells (2 × 10^5^ cells/mL) were seeded in 96-well plates for monitoring all experimental conditions including melatonin pretreatment (100 μM) and insulin stimulation (1 μM), separately. Next, culture medium was replaced with serum-free medium, and 100 μL of 3-(4,5-dimethylthiazol-2-yl)-2, 5-diphenyltetrazolium bromide (MTT) (Sigma-Aldrich, St.Louis, MO, USA) solution (5 mg/mL in PBS) was added to each well. After incubation for 1 h, medium was removed and dimethyl sulfoxide was added to each well to solubilize the purple formazan product of MTT reaction. The supernatant from each well was analyzed using a microplate reader at 570 nm (Labsystems Multiskan MCC/340; Fisher Scientific, Pittsburgh, PA, USA). All experiments were repeated three times. Cell viability in medium of non-treated cells was considered 100%. 

### 2.3. Reverse Transcription-PCR

To examine the mRNA expressions of cleaved Poly [ADP-ribose] polymerase 1 (cleaved PARP), p53, Bax, phosphorylated eukaryotic initiation factor 2 alpha (p-eIF2α), activating transcription factor 4 (ATF4), C/EBP homologous protein (CHOP), and phosphorylated inositol requiring kinase 1 alpha (*p-IRE1*), reverse transcription-PCR (RT-PCR) was performed using each primer. Briefly, samples were lysed with Trizol reagent (Invitrogen), and total RNA was extracted according to the manufacturer’s protocol. PCR was performed using the following primers (5′ to 3′): cleaved PARP (F): AGG CCC TAA AGG CTC AGA AT, (R): CTA GGT TTC TGT GTC TTG AC, p53 (F): CTG CCC TCA ACA AGA TGT TTT G , (R): CTA TCT GAG CAG CGC TCA TGG , Bax (F): AAG AAG CTG AGC GAG TGT, (R): GGA GGA AGT CCA ATG TC, p-eIF2α (F): ACG CTT TGG GGC TAA TTC TT, (R): TCT GGG CTT TTC TTC CAC AC, ATF4 (F): GTC CTA TCT GGG GTC TCC TC, (R): TAC CTA GTG GCT GCT GTC TT, CHOP: (F): AGA ACC AGG AAA CGG AAA CAG A (R): TCT CCT TCA TGC GCT GCT TT, *p-IRE1* (F): GCT GTG GAG ACC CTA CGC TAT , (R): TCG ATG TTT GGG AAG ATT GTT AG, and glyceraldehyde-3-phosphate dehydrogenase (GAPDH) (F): ACA GTC CAT GCC ATC ACT GCC, (R): GCC TGC TTC ACC ACC TTC TTG. PCR products were electrophoresed in 1.5% agarose gels and stained with ethidium bromide. All experiments were repeated three times.

### 2.4. Quantitative Real Time-PCR

To examine the mRNA expression of sliced X-box binding protein 1 (XBP1) in cells under IR conditions, quantitative real time-PCR (qPCR) was performed. Total cellular RNA was extracted from the cells using Trizol reagent (Invitrogen, Carlsbad, CA, USA) according to the manufacturer instructions. Poly (A) was added using poly (A) polymerase (Ambion, Austin, TX, USA). One Step SYBR^®^ Prime Script TM RT-PCR Kit II (Takara, Japan) was used to conduct qPCR. PCR was performed using the following primers (5′ to 3′); sliced XBP1 (F): CTG AGT CCG AAT CAG GTG CAG, (R): ATC CAT GGG GAG ATG TTC TGG, β-actin (F): TCT GGC ACC ACA CCT TCT A, (R): AGG CAT ACA GGG ACA GCA C. The expression of each of the factors was assessed using an ABI prism 7500 Real-Time PCR System (Life Technologies Corporation, Carlsbad, CA, USA) and analyzed with comparative Ct quantification. β-actin was amplified as an internal control. The values were presented by relative quantity (RQ). All experiments were repeated three times.

### 2.5. Western Blot Analysis

SH-SY5Y cells were washed with PBS and harvested together. Cell pellets were lysed with cold radioimmunoprecipitation assay buffer (Sigma-Aldrich, St. Louis, MO, USA). The lysates were centrifuged at 13,000 rpm for 20 min at 4 °C to produce whole-cell extracts. Cellular proteins (30 μg) were separated on a 10% sodium dodecyl sulfate-polyacrylamide gel (SDS-PAGE) and transferred onto a polyvinylidene difluoride membrane. Blocking with skimmed milk prepared in Tris-buffered saline with Tween^TM^ 20 detergent (TBST) (20 nM Tris (pH 7.2) and 150 mM NaCl, 0.1 % Tween^TM^ 20) was performed for 1 h at room temperature. Immunoblots were then incubated for 15 h at 4 °C with primary antibodies that detect cleaved PARP (1:1000, Abcam, Cambridge, MA, USA), p-eIF2α (1:1000, Cell Signaling, Danvers, MA, USA), and β-actin (1:1000; Millipore, Billerica, MA, USA). Blots were then incubated with each secondary antibody (Abcam, Cambridge, MA, USA) for 1 h 30 min at room temperature. Blots were visualized using ECL solution (Millipore, Billerica, MA, USA).

### 2.6. Immunofluorescence for p-ASK-1 and p-IRE1

SH-SY5Y cells were incubated with the primary antibody overnight at 4 °C. The following primary antibodies were used: anti-goat phosphor apoptosis signal regulating kinase 1 (*p-ASK1*) (1:200, Santa Cruz Biotechnology, Santa Cruz, CA, USA) and anti-goat phosphor-IRE1 (*p-IRE1*) (1:200, Cell Signaling, Danvers, MA, USA). The primary antibody was then removed, and the cells were washed three times for 3 min with PBS. The cells were incubated with second antibody for 1 h 30 min at room temperature. The cells were then washed again three times for 3 min with PBS, followed by counterstaining with 1 μg/mL of 4′,6-diamidino-2-phenylindole (DAPI, 1:200, Invitrogen) for 15 min at room temperature. The cells were imaged using a Zeiss LSM 700 confocal microscope (Carl Zeiss, Thornwood, NY, USA).

### 2.7. Statistical Analysis

Statistical analyses were performed using SPSS ver.22.0 software (IBM Corp., Armonk, NY, USA). Results were expressed as mean ± standard deviation (S.D.). Differences among the groups were determined by one-way analysis of variance (ANOVA) followed by Bonferroni post hoc multiple comparison tests. A *p*-value less than 0.05 was considered statistically significant.

## 3. Results

### 3.1. Melatonin Increases SH-SY5Y Cell Viability under IR Conditions

**Figure 1:**
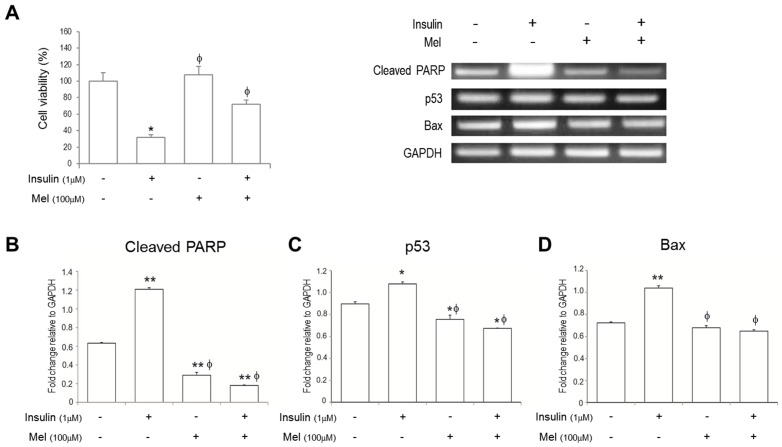
Melatonin alleviates insulin resistance (IR)-induced SH-SY5Y neuronal cell death signaling. (**A**) Cell viability was assessed by 3-(4,5-dimethylthiazol-2-yl)-2, 5-diphenyltetrazolium bromide (MTT) assay. The mRNA levels of (**B**) cleaved PARP; (**C**) p53; and (**D**) Bax were measured by reverse transcription PCR. Data are expressed as mean ± standard error and each experiment included three repeats per conditions. * *p* < 0.05, ** *p* < 0.01 compared with non-stimulated control; ^φ^ *p* < 0.05 compared with insulin stimulated cells. Mel: melatonin pretreatment for 24 h before insulin stimulation.

The SH-SY5Y cell viabilities were measured using MTT assay (Figure 1A). The cell viability was markedly reduced by insulin stimulation compared with non-stimulated control. On the other hand, the decreased cell viability by insulin stimulation was recovered when treated with melatonin. 

### 3.2. Melatonin Treatment Alleviates IR-Induced Neuronal Cell Death Signaling 

**Figure 2:**
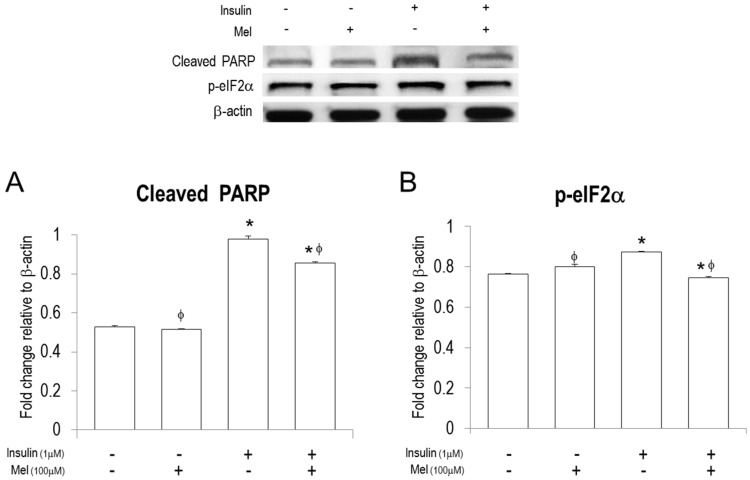
Melatonin regulates protein levels of cleaved PARP and p- p-eIF2α in the insulin stimulated SH-SY5Y neuronal cells. Protein levels of (**A**) cleaved PARP and (**B**) activation of eIF2α, (p-eIF2α) were measured by western blot analysis. Data are expressed as mean ± standard error and each experiment included three repeats per conditions. * *p* < 0.05 compared with non-stimulated control; ^φ^ *p* < 0.05 compared with insulin stimulated cells. Mel: melatonin pretreatment for 24 h before insulin stimulation.

To investigate if neuronal cell death signaling under insulin stimulation is regulated by melatonin treatment, we measured mRNA expressions of cleaved PARP, p53, and Bax using RT-PCR (Figure 1B–D). mRNA expressions of cleaved PARP, p53, and Bax were significantly increased under IR condition. Melatonin treatment dramatically attenuated mRNA expressions of cleaved PARP and p53 in the cells stimulated by insulin or not. Furthermore, the attenuated levels by melatonin treatment were much lower than those in non-stimulated controls. In addition, the increased mRNA levels of Bax under IR condition were significantly attenuated by melatonin treatment. In addition, western blot assay shows that melatonin treatment significantly attenuated the insulin-induced expression of cleaved PARP (Figure 2A). 

### 3.3. Melatonin Treatment Regulates IR-Induced ER Stress Signaling in Neuronal Cells 

**Figure 3:**
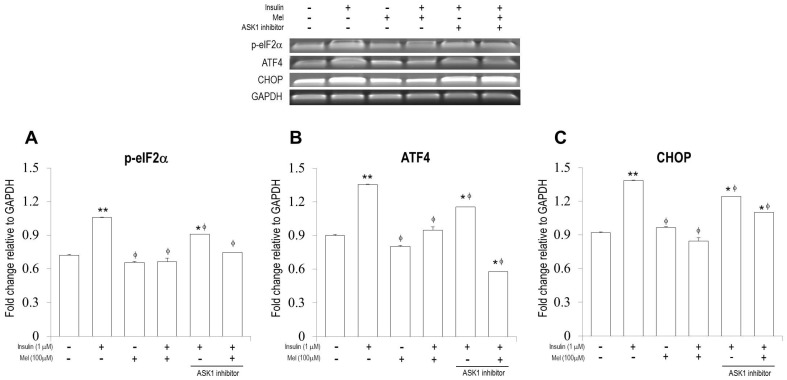
Melatonin regulates IR-induced endoplasmic reticulum (ER) stress signaling (p-eIF2α, ATF4, and CHOP) in the SH-SY5Y neuronal cells. The mRNA levels of (**A**) activation of eIF2α, (p-eIF2α); (**B**) ATF4, and (**C**) CHOP were measured by reverse transcription PCR. Data are expressed as mean ± standard error and each experiment included three repeats per conditions. * *p* < 0.05, ** *p* < 0.01 compared with non-stimulated control; ^φ^ *p* < 0.05 compared with insulin stimulated cells. Mel: melatonin pretreatment for 24 h before insulin stimulation; *ASK1* inhibitor: NQDI-1 600 nM treatment for 2 h before sampling.

**Figure 4:**
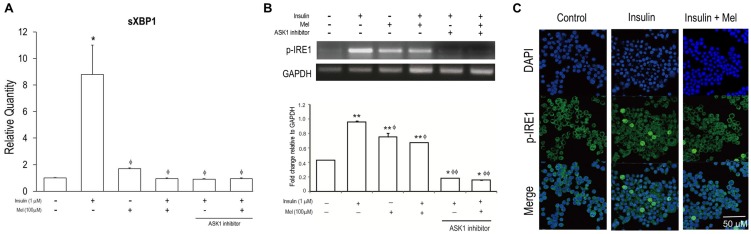
Melatonin alleviates IR-induced ER stress signaling (sXBP1 and *p-IRE1*) in the SH-SY5Y neuronal cells. (**A**) The mRNA levels of sXBP1 were measured by quantitative real time PCR; (**B**) The mRNA levels of activation of IRE1 (*p-IRE1*) were measured by reverse transcription PCR. Data are expressed as mean ± standard error and each experiment included three repeats per conditions. * *p* < 0.05, ** *p* < 0.01 compared with non-stimulated control; ^φ^ *p* < 0.05, ^φφ^ *p* < 0.001 compared with insulin stimulated cells; (**C**) *p-IRE1* was visualized by immunofluorescence staining using confocal microscopy analysis. *p-IRE1* is represented by green staining, nuclear DNA is indicated by 4′,6-diamidino-2-phenylindole (DAPI) staining (blue color), and the combined images are presented. Mel: melatonin pretreatment for 24 h before insulin stimulation; *ASK1* inhibitor: NQDI-1 600 nM treatment for 2 h before sampling.

To investigate if melatonin treatment regulates ER stress in neuronal cells under insulin stimulation, we performed RT-PCR, qPCR, and Immunofluorescence analysis (Figure 3 and Figure 4). As shown in Figure 3A–C, mRNA expressions of p-eIF2α, ATF4, and CHOP were significantly increased by insulin stimulation, but the increased expressions were significantly attenuated by melatonin treatment. Similar patterns were observed when *ASK1* signaling was inhibited. Western blot assay also showed that melatonin treatment significantly attenuated the insulin-induced expression of p-eIF2α (Figure 2B). 

It can be observed in Figure 4 that significantly increased mRNA expressions of sXBP1 under IR condition were dramatically attenuated when melatonin was treated together with or without *ASK1* inhibitor. As shown in Figure 3A, mRNA expressions of sXBP1 were dramatically increased by insulin stimulation, but the increased expressions were significantly attenuated by melatonin treatment together with or without *ASK1* inhibitor. mRNA expression of *p-IRE1* was also significantly increased by insulin stimulation, and attenuated by melatonin treatment (Figure 4B). Interestingly, when *ASK1* signaling was inhibited, mRNA expressions of *p-IRE1* stimulated by insulin treated with melatonin or not were dramatically attenuated. In addition, immunofluorescence analysis confirmed that *p-IRE1* induced by insulin stimulation was significantly suppressed by melatonin treatment (Figure 4C). 

### 3.4. Melatonin Attenuates the Activation of ASK1 under IR Condition

**Figure 5:**
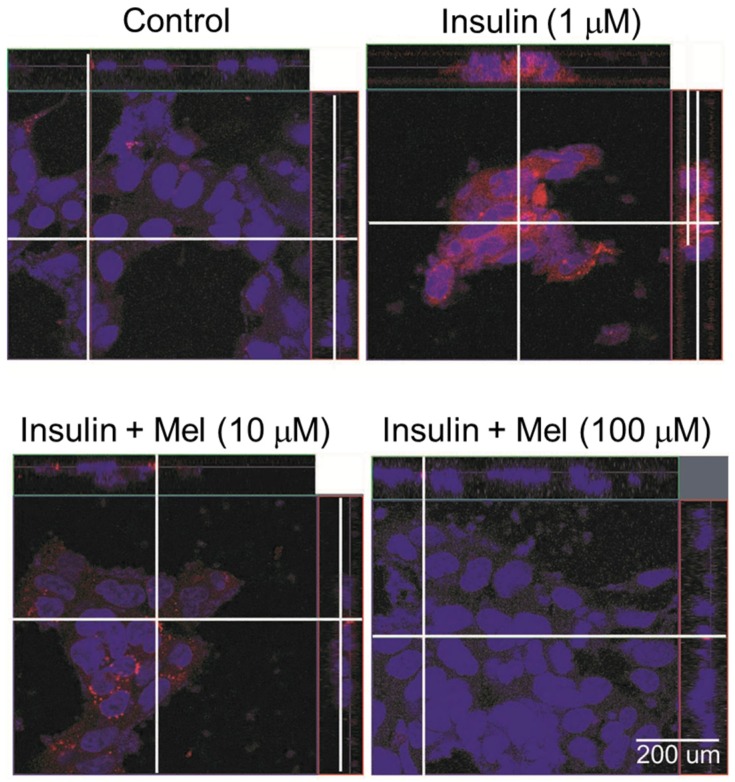
Melatonin attenuates the activation of *ASK1* in insulin stimulated SH-SY5Y cells. Immunofluorescence staining was performed to check the activation of *ASK1* (*p-ASK1*). Confocal microscopy analysis was performed to visualize *p-ASK1*. SH-SY5Y cells were pretreated with melatonin for 24 h before insulin stimulation. *p-ASK1* is represented by red staining, nuclear DNA is indicated by DAPI staining (blue color), and the combined images are presented; Control: non-treated cells, Mel: melatonin.

Immunofluorescence analysis was conducted to check if melatonin regulates the activation of *ASK1* under IR condition (Figure 5). *ASK1* was markedly phosphorylated by insulin stimulation, but the activation was dose-dependently attenuated by melatonin treatment (10 μM and 100 μM). 

## 4. Discussion

The present study shows that melatonin modulates neuronal cell death induced by ER stress under IR condition. Insulin-induced mRNA or protein expressions of cell death signaling markers such as cleaved PARP, p53, and Bax, as well as the ER stress markers such as p-eIF2α, ATF4, CHOP, sXBP1, and *p-IRE1* were significantly attenuated by melatonin treatment. In addition, the regulatory effect of melatonin on insulin-induced ER stress in neuronal cells was associated with *ASK1* signaling. These results suggested that melatonin may ameliorate IR-induced neuropathogenesis via the regulation of ER stress.

Recent studies reported that IR is closely linked to hippocampus cognitive dysfunction [3,4,5,6,49], and is a crucial factor for determining the processing of neuropathogenesis [50,51]. It may be associated with the dysregulation of cellular homeostasis [9] and ER stress, which triggers cell death signaling [10,11]. The ER is highly sensitive to stresses, which reduce the protein folding capacity of the ER, thereby resulting in the accumulation and aggregation of unfolded proteins [12]. The aggregation of proteins is toxic to cells and consequently, associated with various pathophysiological conditions such as DM, ischemia, and neurodegenerative disease [52,53,54,55,56,57,58,59]. In our study, we found that mRNA and protein expressions of ER stress markers (p-eIF2α, ATF4, CHOP, *p-IRE1*, and sXBP1) [13,14,15] in the SH-SY5Y neuronal cells were significantly increased by insulin stimulation. mRNA expressions of apoptotic cell death markers (cleaved PARP, Bax, and p53) [60,61,62] were also significantly increased by insulin stimulation. In addition, the imaging analysis demonstrated that IR condition dramatically activated ASK. As mentioned above, under stressful conditions, IR might sequentially activate the ER stress receptors, thereby triggering ER stress-induced cell death signaling: for example, PERK, one of the ER stress receptors, might phosphorylate eIF2α, which translocated ATF4 into nucleus, and then increased the gene transcription such as CHOP to restore ER homeostasis, thereby regulating ER stress mediated apoptosis [12,13,14,15]. In addition, IRE, another ER stress receptor might be activated by IR stress, and spliced XBP1, one of the ER chaperones, which was translocated into the nucleus, and then modulates the gene transcription involved in protein degradation [20,21]. Under the ER stress, the activated IRE makes complex with TRAF2, and then recruits ASK1, thereby triggering cell death [24,25,26,27,28]. 

As mentioned above, melatonin and its metabolites were reported to modulate inflammation, cell survival, and apoptosis in various pathophysiological conditions [51,52,53,54,55] through their capacity as potent antioxidants and free radical scavengers [56,57]. According to previous reports [48,63,64,65], melatonin supplementation significantly improves antioxidant status by increasing the activity of antioxidant enzymes (i.e., superoxide dismutase, glutathione peroxidase, catalase, etc.) in T2DM and obesity [48,63,64,65]. These enzymes also contain antioxidant minerals such as Zn, Mn, Fe, Se, Cu, etc. [48,63,64,65]. In the present study, we found that insulin-induced mRNA or protein expressions of the ER stress markers and cell death signaling markers in the neuronal cells were significantly attenuated by melatonin treatment, and the activation of *ASK1* induced by insulin stimulation was also dose dependently attenuated by melatonin treatment. Our results might be supported by previous reports [66,67,68,69]. Melatonin can significantly inhibit ER stress by reducing XBP1 splicing, the target of the IRE1 pathway [67,68], JNK phosphorylation [68], and insulin receptor substrate-1 (IRS-1) phosphorylation [69], thereby ameliorating insulin sensitivity. Melatonin was also reported to attenuate tunicamycin-induced ER stress by restoring insulin stimulated Akt phosphorylation and IRS-1 tyrosine phosphorylation, and reducing the IRE-1/JNK phosphorylation and IRS-1 serine phosphorylation [66]. Tunicamycin was known to induce IR through the inhibition of insulin-stimulated Akt phosphorylation, IRE-1/JNK phosphorylation, and IRS-1 serine phosphorylation [66]. In regard to this point, our results demonstrated that melatonin, through its antioxidant properties, modulates the ER stress-induced neuronal cell death under IR condition. Interestingly, our study also shows that when *ASK1* signaling was inhibited, mRNA expressions of *p-IRE1* that were stimulated by insulin treated with melatonin or not were dramatically attenuated. ASK1, which triggers apoptotic cell death, is activated by the IRE1–TRAF2 complex formed under the ER stress [24,25,26,27,28]. According to previous reports [28,70], *ASK1* overexpression induces apoptosis in various cell types, whereas neurons from the *ASK1* knock-out mice exhibited resistance to lethal ER stress. Based on our results and the previous reports, we assumed that *ASK1* signaling is important in ER stress-induced cell death. Further study is needed to identify the precise mechanism on the role of melatonin in *ASK1* signaling in neuronal cells 

In addition, ER stress was reported to induce autophagy [71]. Autophagy is a dynamic process which promotes self-digestion of misfolded proteins and damaged organelles in the cells [35]. Activation of autophagy signaling was observed in obese people [72], and is critical to regulate IR-induced ER stress in diabetic patients [73]. For example, autophagy captures and ubiquitinates inflammasome, thereby recruiting *LC3* as well as *beclin 1*, marker proteins for autophagy detection [74,75,76]. In our study, we did not directly measure the expressions of autophagic cell death related factors such as *belcin1* and *LC3*, but we observed that mRNA expressions of sXBP1 and CHOP, which are involved in autophagic response, were significantly increased by insulin stimulation, and dramatically attenuated by melatonin treatment. sXBP1 was reported to directly bind to the promoter of *beclin 1* in the nucleus, and promotes an autophagic response [77]. CHOP is also known to promote autophagy gene expression (i.e., *LC3*, *NIX*, *NBR1*), and its capacity to dephosphorylate eIF2α is implicated in the initiation of autophagy.

## 5. Conclusions

Taken together, this study demonstrated that melatonin modulates neuronal cell death signaling induced by ER stress under IR condition, and it may be related to the suppression of *ASK1* activation. Furthermore, melatonin may be a potential solution in ameliorating IR associated neuropathogenesis and cognitive decline. Further studies are needed to elucidate the mechanism though which melatonin affects IR-induced ER stress autophagic signaling in order to better understand the neuronal cell damage caused by IR.
